# Case report: Surgical management of a rare right coronary artery fistula with a giant pseudoaneurysm compressing the pulmonary vein in a young patient

**DOI:** 10.3389/fcvm.2023.1233873

**Published:** 2023-09-13

**Authors:** Weijie Tang, Kun Xiang, Huatao Zhou, Yichen Li, Jinfu Yang, Jijia Liu, Chengming Fan

**Affiliations:** Department of Cardiovascular Surgery, The Second Xiangya Hospital, Central South University, Changsha, China

**Keywords:** coronary artery fistula, cardiac surgery, pseudoaneurysm, reconstruction, pulmonary vein

## Abstract

Congenital coronary artery fistula (CAF) represents a remarkable rarity within the realm of cardiovascular anomalies, characterized by an aberrant connection between coronary arteries and either cardiac chambers or major vessels. Clinical manifestations of CAFs often remain unspecified or may even be entirely absent, posing diagnostic challenges. Notably, patients harboring substantial CAFs may exhibit symptoms such as palpitations, chest tightness, and dyspnea. Although right-sided congenital CAFs are relatively prevalent, the occurrence of a CAF accompanied by a colossal pseudoaneurysm imposing compression upon the pulmonary vein is an exceedingly rare phenomenon. This exceptional case report delineates a singular fistula originating from the right coronary artery, extending its course to the right atrium, and remarkably featuring a substantial pseudoaneurysm exerting compression upon the right superior pulmonary vein. Therapeutic intervention encompassed surgical closure of the proximal artery and excision of the pseudoaneurysm, underscoring the complexity and criticality of managing such intricate cardiac anomalies to ensure optimal patient outcomes.

## Introduction

Congenital coronary artery fistula (CAF) is an extremely rare cardiovascular anomaly that is defined as an abnormal connection between the coronary arteries and cardiac chambers or any segment of the systemic or pulmonary circulation, accounting for approximately 0.25%–0.4% of congenital cardiac abnormalities (1–3). Furthermore, CAF accounts for 48.7% of all congenital coronary artery abnormalities and affects 0.002% of the population worldwide (4, 5). Fistulas can be large (>250 mm) and usually increase in size over time (6). Oral fistulas have been reported in the pulmonary artery, right ventricle, right atrium (RA), and left ventricle in 28%, 27%, 22%, and 10% of patients with CAF, respectively (7). The coronary artery or its branches produce a feeding artery that is usually dilated and tortuous. Of note, multiple feeding arteries can drain via a fistula; multiple fistulas can be created from one artery. Patients with small fistulas are usually asymptomatic; however, patients with large fistulas may present with dyspnea, fatigue, palpitations, angina, or congestive cardiac failure (8). The American College of Cardiology/American Heart Association suggests percutaneous or surgical closure of large fistulas as the treatment (9). Herein, we report an extremely rare fistula originating from the right coronary artery (RCA) and extending to the RA with a giant pseudoaneurysm that compressed the right superior pulmonary vein in a young patient. We successfully treated the patient by performing surgical closure of the proximal artery and excising the pseudoaneurysm.

## Case presentation

**Figure 1:**
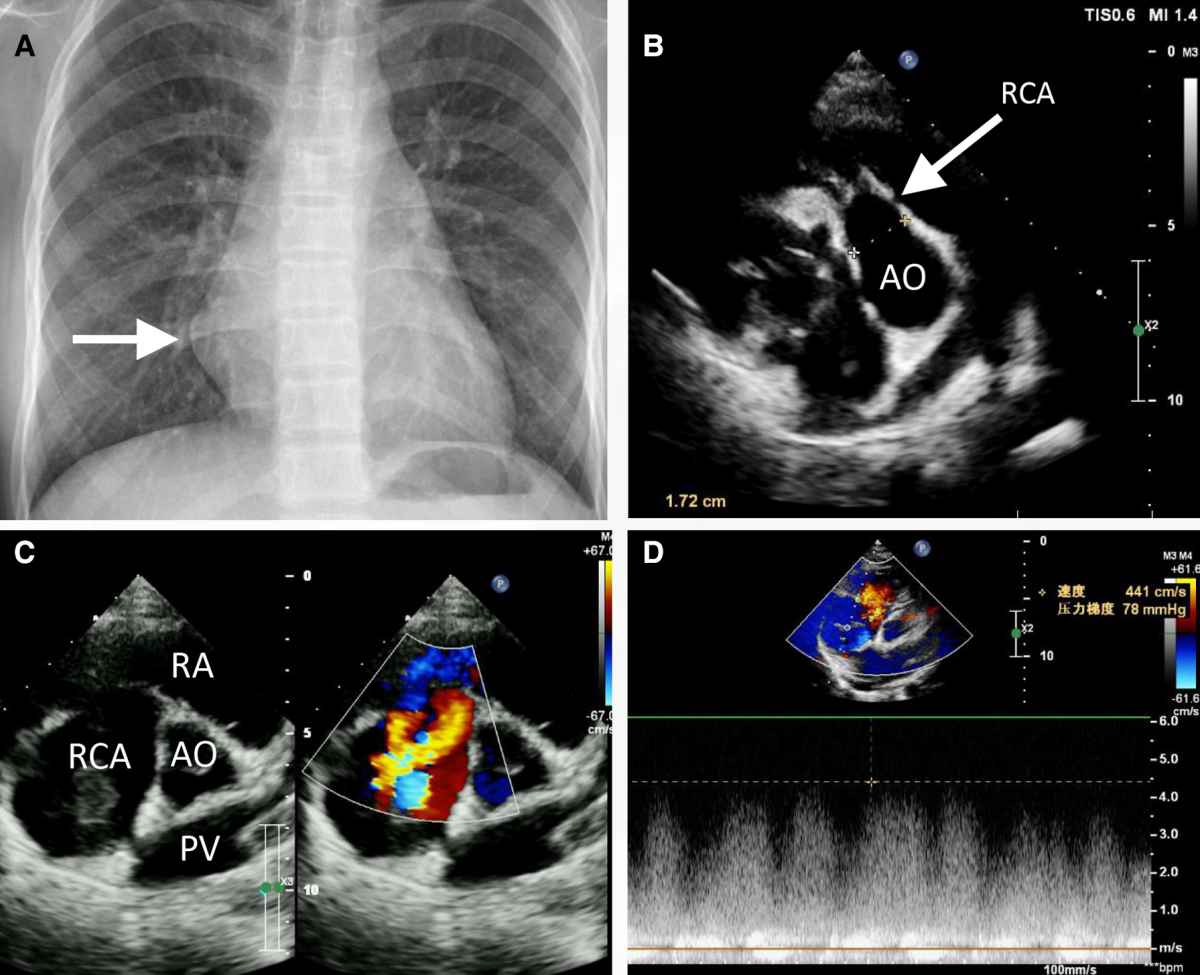
Preoperative chest radiography (**A**) and transthoracic echocardiogram (**B, C**) revealed the partially enlarged heart shadow (**A**, arrow), right coronary artery enlargement (**B**, arrow), pseudoaneurysm (**C**), and right coronary artery to right atrium fistula (**C, D**). AO, ascending aorta; PV, pulmonary vein; RA, right atrium; RCA, right coronary artery.

**Figure 2:**
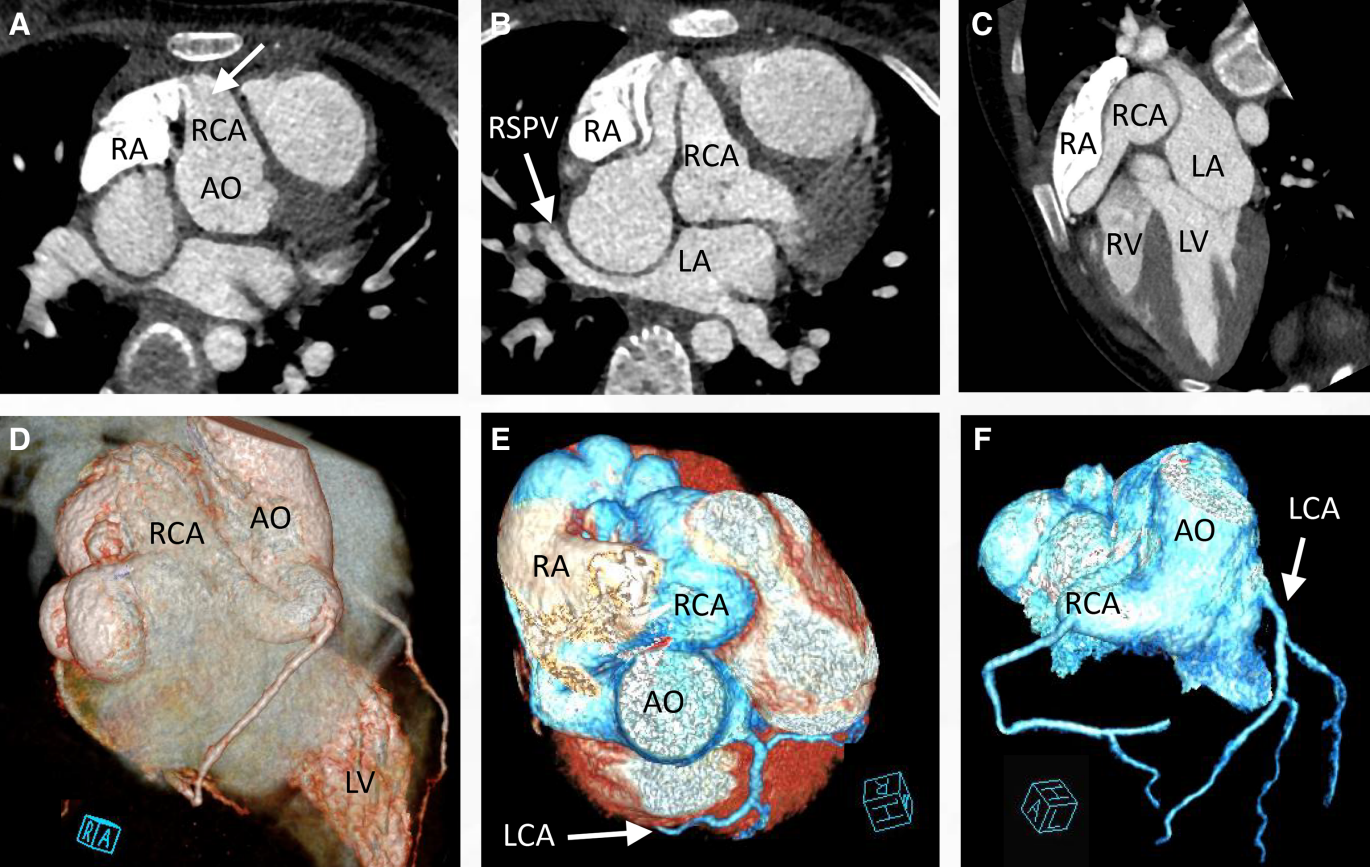
Cardiac computed tomography angiography preoperatively confirmed that the right coronary artery was significantly enlarged (**A**, arrow) to form a pseudoaneurysm (**B**), with the right superior pulmonary vein remarkably compressed (**B, C**, arrow). Three-dimensional reconstruction of the cardiac CTA indicated that the enlarged right coronary artery was tortuous (**D, E**) and the left coronary artery functioned normally (**F**, arrow). CTA, computed tomography angiography.

An 11-year-old female patient (weight: 44.5 kg; height: 161 cm) was admitted to the hospital due to a persistent palpitation episode lasting approximately 2 h, which spontaneously subsided following a period of rest. The palpitations had been recurring over the course of 3 months and occurred without an identifiable trigger. The patient reported no other accompanying symptoms. Physical examination upon admission detected a continuous systolic murmur emanating from the apical region of the heart and precordium. An electrocardiogram revealed a normal rhythm, while a chest x-ray exhibited an enlarged RA (Figure 1A). A two-dimensional echocardiogram provided further insights, revealing an enlarged RA and indicating an uneven and widened inner diameter of the RCA measuring 16 mm. The patient's preoperative left ventricular diameter was measured at 43 mm, and the right ventricular diameter was 30 mm. The RCA was observed originating from the right coronary sinus, curving rightward and posteriorly before turning leftward to connect with the lateral wall of the RA (Figure 1B). Color Doppler imaging corroborated the presence of a left-to-right shunt during both systole and diastole (Figures 1C,D). The preliminary diagnosis established a congenital CAF originating from the RCA and extending into the RA. Subsequent computed tomography and angiography of the cardiac structures and coronary arteries unveiled significant findings. The RCA displayed thickening and tortuosity, with its largest diameter measuring approximately 3.2 cm, indicative of a pseudoaneurysm. Moreover, the RCA terminated by merging with the lateral wall of the RA, forming an internal fistulous diameter of around 4 mm at the RA connection point. Notably, this anatomical configuration resulted in severe external compression of the right superior pulmonary vein, which exhibited a narrowest diameter of about 4 mm. In contrast, the left coronary artery demonstrated unimpaired function (Figure 2). Consequently, the comprehensive diagnosis encompassed a CAF originating from the RCA and extending to the RA, concomitant with the presence of a substantial pseudoaneurysm inducing compression upon the right superior pulmonary vein.

**Figure 3:**
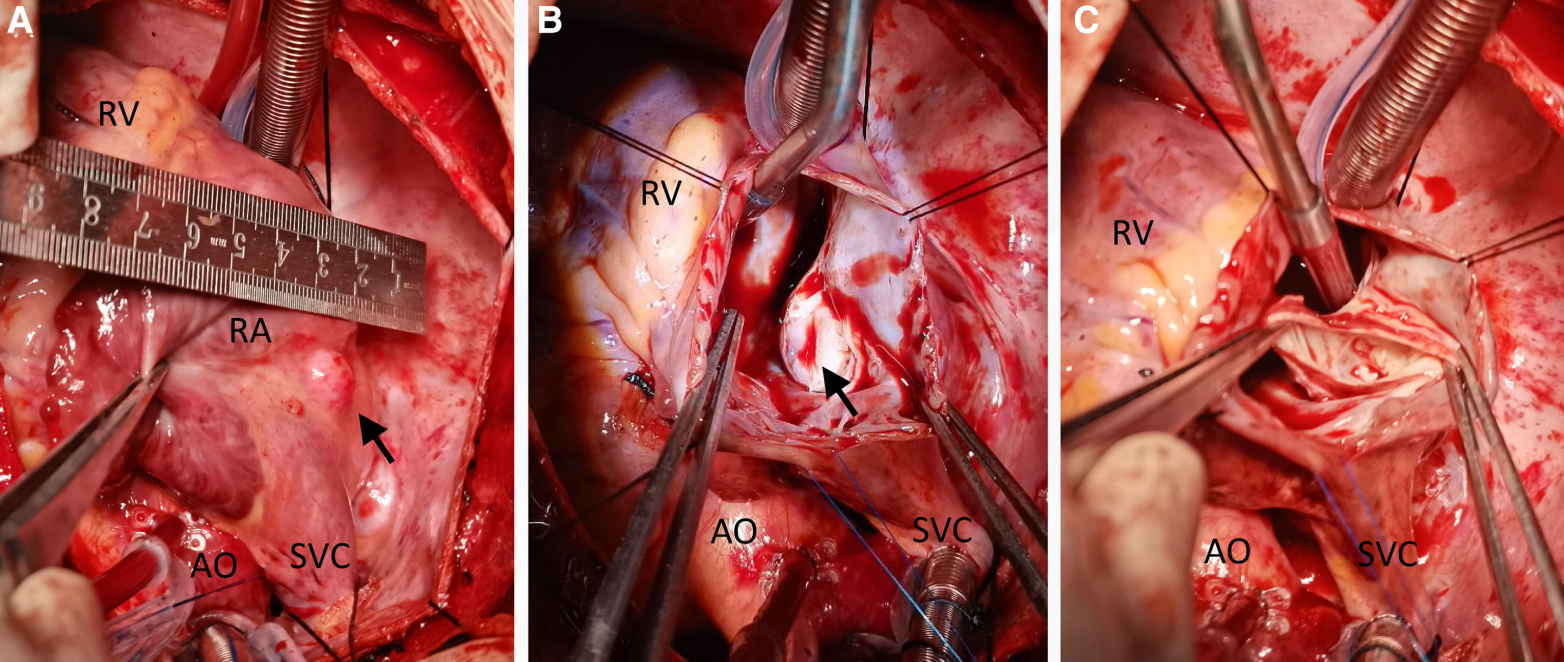
Intraoperative view showing a cystic lesion located beneath the right and above the right superior pulmonary vein (**A–C**). AO, ascending aorta; RA, right atrium; RV, right ventricle; SVC, superior vena cava.

**Figure 4:**
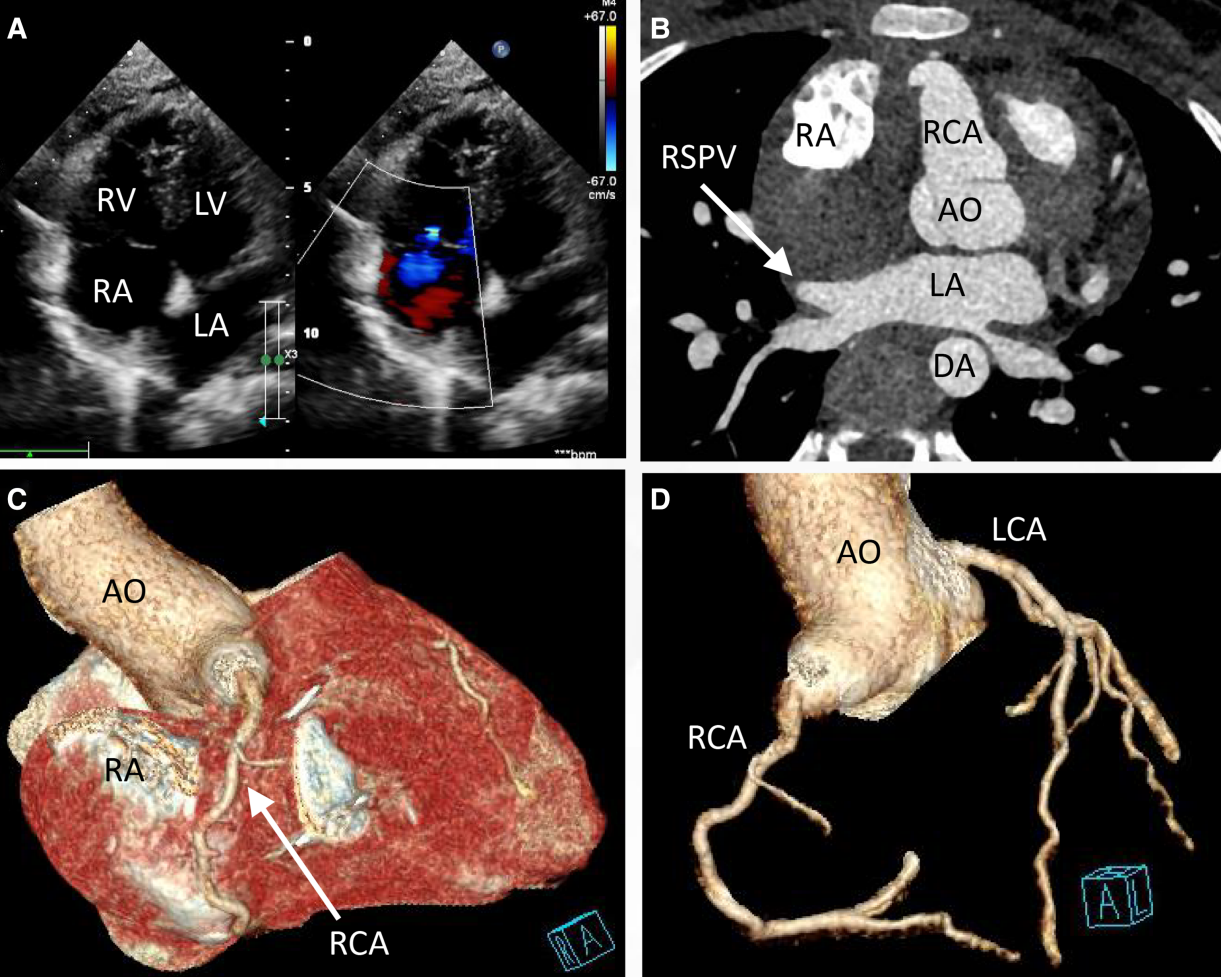
Transthoracic echocardiogram (TTE) and coronary computed tomography angiography at discharge (day 7 postoperatively) indicated that the valves functioned well (**A**) and the right superior pulmonary vein was decompressed (**B**, arrow). The pseudoaneurysm was removed (**C**) No tortuous artery or enlargement was detected in the coronary arteries (**D**).

Engaging in discussions with the patient's parents regarding the optimal treatment approach proved intricate. Although the option of embolization was presented, it was met with refusal due to financial constraints. Instead, the parents sought surgical resection as a viable alternative. Consequently, an open-heart surgery was performed through a median sternotomy with cardiopulmonary bypass but free from aortic cross-clamp and cardioplegic solution infusion. During the surgical intervention, a pronounced enlargement of the RCA pseudoaneurysm, along with multiple fistulas extending into the RA, became evident (Figure 3). The RA's right wall harbored multiple enlarged coronary artery aneurysms, particularly at the site of convergence with the superior vena cava, extending from the RA apex to its lateral wall. To facilitate the procedure, an abnormal branch of the RCA was temporarily ligated using a 10-gauge wire. Within the initial 15 min, the patient exhibited stable cardiac rhythm and blood pressure; however, occlusion occurred irreversibly thereafter. In response, the aneurysm was excised, and proximal artery closure was meticulously performed. The postoperative trajectory remained devoid of significant complications. The surgical procedure itself was characterized by a cardiopulmonary bypass time of 66 min, obviating the need for cardiopulmonary arrest. Concurrent circulation was maintained, supported by mechanical ventilation over a 15-hour period. Post-surgery, the patient's stay in the intensive care unit spanned 20.5 h. A swift recovery ensued, culminating in the patient's discharge on the seventh day following the operation. Subsequent postoperative evaluations, encompassing transthoracic echocardiography and coronary computed tomography angiography, corroborated the successful functioning of the heart valves and coronary artery, with no evidence of stenosis (Figure 4). Furthermore, analysis of cardiac and coronary computed tomography images revealed the relief of pulmonary vein stenosis, evidenced by a narrowing of approximately 10.1 mm at the pulmonary vein’s narrowest point. During the ensuing three-month follow-up period, the patient exhibited a favorable recovery trajectory devoid of complications, ultimately experiencing a resumption of normal activities without the recurrence of symptoms.

## Discussion and conclusion

CAFs are rare heart anomalies, affecting 1 in every 50,000 live births and approximately 0.2% of adults (10). CAF is characterized by inappropriate direct communication of a branch of the main, right, or left coronary artery with the ventricles or major arteries (11). CAF occurs due to abnormal embryological development of the sinusoidal space in the myocardium. In our patient, leakage into the right heart chamber caused a left-to-right shunt, resulting in increased right heart burden and pulmonary blood flow. The artery drains into a low-pressure site; however, the blood tends to flow into the drainage site rather than the smaller arterioles and capillaries of the myocardium; this is known as the “steal phenomenon,” and it may lead to cardiac ischemia (12). More than 50% of these fistulas are congenital and originate from RCA (10).

The clinical manifestations of CAF are unclear and often absent. Large CAFs may be associated with palpitations, chest tightness, dyspnea, and other clinical manifestations. Auscultation detects a continuous murmur, most prominently during diastole, whereas fistulas entering the left ventricle often reveal a diastolic murmur. The symptoms of CAF can be heart murmurs, congestive heart failure, or angina pectoris. Endocarditis can also occur in CAFs that are left untreated (13). Coronary angiography is the most effective and reliable imaging technique for the diagnosis of CAF. Complementary diagnostic methods include transthoracic and transesophageal echocardiography, computed tomography, and magnetic resonance imaging (14). A comprehensive preoperative imaging evaluation holds paramount importance in precisely identifying the localization of congenital coronary artery anomalies, their associated feeding vessels, and intricate spatial relationships with neighboring structures. Leveraging a multimodal imaging approach, coupled with detailed morphological and functional insights, proves instrumental in both preoperative diagnosis and precise localization of these anomalies. This approach yields tangible benefits, notably in curtailing the occurrence of intraoperative and postoperative complications, thereby fostering an enhanced patient prognosis (15, 16). The most important and fatal consequences of CAF are pulmonary hypertension, congestive heart failure, myocardial ischemia, aneurysmal CAF rupture, and infective endocarditis (7, 17).

A conservative approach is recommended due to complaints of spontaneous closure of fistulas and the presence of asymptomatic or slightly symptomatic patients (6, 18). Nevertheless, the majority of CAF treatments are effective, including minimally invasive and surgical treatments. The surgical treatment is successful in most cases. The possible complications of CAFs are acute thrombosis, distal embolization of RCA, compression of the heart, and rupture. All symptomatic patients, such as our patient, should be treated with surgical repair because the risk of surgery is substantially lower than that of potentially fatal sequelae. Although surgical therapy is favored in most cases, interventional closure of larger fistulas is also an option for treatment (18–21). Deliberating the optimal surgical approach, our consideration initially inclined towards a small right subcostal incision combined with transthoracic pericardial access, theoretically offering reduced invasiveness to the patient. However, our decision pivoted on a series of factors: the patient's age of 11 years, the relatively lower positioning of the coronary aneurysm and coronary fistula, and the prospect of employing extracorporeal circulation as part of the preoperative assessment. Concerns emerged regarding the potential limitations of the compact subcostal incision in fully exposing the operative field, potentially influencing surgical outcomes. In light of these considerations, a median thoracotomy emerged as the chosen approach. This decision aimed to ensure comprehensive visualization of the surgical site, compensating for potential limitations posed by the subcostal alternative. Additionally, analogous reasoning prompted the rejection of a small incision at the lower end of the sternum. This option was deemed less favorable due to its potential to hinder the exposure of the aortic root, and further, its potential inadequacy in fully revealing the coronary aneurysm and coronary fistula. In pursuit of an encompassing and effective surgical strategy, the comprehensive evaluation of these factors led us to opt for a median sternum incision.

Our case report describes a rare case of CAF with grape-like beading alterations, wherein we performed surgical closure of the proximal artery and excised the distal aneurysm. Before ligation, we evaluated whether the artery was a sinus node artery; we ligated it for 10 min to observe its effect on heart rate. The heartbeat remained unaltered; thus, considering that it was a branch of RCA, we ligated and resected it. For similar cases in the future, it is possible to consider whether the aneurysm is a sinus node artery preoperatively or try to ligate it. If the heart rate is not affected, it can be directly surgically ligated with an excellent prognosis.

## Data Availability

The raw data supporting the conclusions of this article will be made available by the authors, without undue reservation.
